# Propensity Scoring after Multiple Imputation in a Retrospective Study on Adjuvant Radiation Therapy in Lymph-Node Positive Vulvar Cancer

**DOI:** 10.1371/journal.pone.0165705

**Published:** 2016-11-01

**Authors:** Christine Eulenburg, Anna Suling, Petra Neuser, Alexander Reuss, Ulrich Canzler, Tanja Fehm, Alexander Luyten, Martin Hellriegel, Linn Woelber, Sven Mahner

**Affiliations:** 1 Medical Statistics and Decision Making, Department for Epidemiology, University Medical Center Groningen, Groningen, The Netherlands; 2 Department of Medical Biometry and Epidemiology, University Medical Center Hamburg-Eppendorf, Hamburg, Germany; 3 KKS Philipps University Marburg, Marburg, Germany; 4 Dept. of Gynecology and Obstetrics, University of Dresden, Dresden, Germany; 5 Dept. of Gynecology University Medical Center Duesseldorf, Duesseldorf, Germany; 6 Dept. of Gynecology and Obstetrics, University Hospital Tuebingen, Tuebingen, Germany; 7 Dept. of Gynecology, Obstetrics and Gynecologic Oncology, Klinikum Wolfsburg, Wolfsburg, Germany; 8 Dept. of Gynecology, Georg-August-University Goettingen, Goettingen, Germany; 9 Department of Gynecology and Gynecologic Oncology, University Medical Center Hamburg-Eppendorf, Hamburg, Germany; 10 Department of Gynecology and Obstetrics, Ludwig-Maximilians-University, Munich, Germany; University Hospital Jena, GERMANY

## Abstract

Propensity scoring (PS) is an established tool to account for measured confounding in non-randomized studies. These methods are sensitive to missing values, which are a common problem in observational data. The combination of multiple imputation of missing values and different propensity scoring techniques is addressed in this work. For a sample of lymph node-positive vulvar cancer patients, we re-analyze associations between the application of radiotherapy and disease-related and non-related survival. Inverse-probability-of-treatment-weighting (IPTW) and PS stratification are applied after multiple imputation by chained equation (MICE). Methodological issues are described in detail. Interpretation of the results and methodological limitations are discussed.

## Introduction

One of the pertinent challenges in estimating causal treatment effects from observational data is to control for confounding bias. The lack of randomization can lead to systematic differences between treated and untreated subjects. In this case, observed differences in outcome cannot securely be attributed to treatment exposure. Propensity scoring (PS) is the established statistical approach to reduce bias resulting from imbalanced measured covariate distributions across treatment groups [1–5]. The propensity score (PS) e(x_i_) for a subject i is the probability that the subject receives the treatment Z_i_, given its individual vector of covariates x_i_, e(x_i_) = P(T_i_ = 1|x_i_). Z_i_ = 1 applies if subject i receives the treatment, otherwise Z_i_ = 0. Various PS methods exist including PS matching[2], PS stratification[6] PS covariate adjustment[7] and inverse-probability-of-treatment-weighting (IPTW)[8]. All PS models are very sensitive to missing values, which are regularly encountered in retrospective studies. Patients or alternatively covariates with missing data have to be excluded from the analysis. Different approaches to solve the problem of missing values in PS analyses have been studied[9–14]. The multiple-imputation-by-chained-equations (MICE) has been demonstrated to be an appropriate method to deal with missing values, if they are missing at random[13–16]. With this method, missing values are replaced by repeatedly drawn values from conditional probability distributions.

The results of the primary analysis and of one propensity score approach using available data of the AGO-CaRE 1 (**A**rbeitsgemeinschaft **G**ynäkologische **O**nkologie—**C**hemo- **a**nd **R**adiotherapy in **E**pithelial Vulvar Cancer) study were reported in a medical companion paper[17]. We re-analyzed the data, containing lymph-node positive vulvar cancer patients, of which a subgroup was treated with adjuvant radio(chemo)therapy. Associations with mortality from vulvar cancer (disease-related death (DRD)) and death from other / unknown causes (DOC) were analyzed as competing risks. In the present work, the methodology of data analysis using multiple imputation and propensity scoring to estimate causal effects from observational data is shown in detail and considerations about methodological issues are disclosed. The specific focus of this work is the detailed description and discussion of the applied statistical methodology. The use of the applied techniques are opposed to other potential techniques. Advantages and disadvantages are discussed.

## Patients

In the AGO-CaRE 1 study, 1618 patients with advanced vulvar cancer (FIGO stage ≥ IB [UICC staging 2006]) treated between 1998 and 2008 were retrospectively collected[13]. In the present analysis, a subgroup of 346 patients with lymph-node involvement, age ≤90 years and documented follow-up status were included. Of these patients, 182 (52.6%) were treated with adjuvant radiotherapy, whereas 164 (47.4%) did not receive adjuvant radiotherapy.

## Ethical Approval and Informed Consent

The study protocol was approved by local ethics committees at each center [leading vote: Hamburg (reference number PV3658)] and registered with clinicaltrials.gov (NCT01304667). Patients provided general written informed consent to access their medical records for scientific analysis at first contact with the respective study center. All procedures performed in studies involving human participants were in accordance with the ethical standards of the institutional and/or national research committee and with the 1964 Helsinki declaration and its later amendments or comparable ethical standards.

## Statistical Methods

### Multiple imputation

The MICE approach is an established imputation method creating multiple complete data sets in which the missing values are replaced by estimates from a specified regression model using the observed data[13;15;16]. The procedure assumes the missing data to be missing at random, which means that the probability that a value is missing only depends on the measured data.

With these multiply-imputed data sets, estimation is possible without omitting covariates or individuals with missing values. Let x_1_,…,x_k_ be the k variables to be considered, with some or all of them having missing values. In the first step, all missing values are replaced at random. Then, the first variable with missing values, e.g. x_1_, is regressed on the variables x_2_,…,x_k_. From this estimation model on observed values of x_1_, a prediction of x_1_ is generated, from which the missing values of x_1_ are replaced by simulated drawing. The next variable with missing values x_2_ is regressed on all other variables x_1_,x_3_,…,x_k_, including the imputed values. Again, missing values of x_2_ are replaced by drawings from the posterior predictive distribution of x_2_. The procedure is repeated for all variables with missing values. After completion of such one cycle, the procedure is replicated for ten cycles to create one complete data set with stabilized imputations, X_complete_. It is recommended to generate m = 3–20 data sets $X_{complete}^{\left(1\right)},\ldots ,X_{complete}^{\left(m\right)}$ [10;13;18]. In this analysis, m = 10 complete data sets were generated. Considered variables were those listed in Table 1, except resection margin and lymph node metastasis diameter, as these variables contained too many missing values (62% and 70%, respectively). The different types of variables (continuous, dichotomous, categorical) were accounted for, and implausible values (negative count data, non-existing categories) were avoided[10;13]. To account for possible imbalances of the covariates amongst the treated and untreated patients, MI was conducted for both treatment groups separately[10].

**Table 1:** Patient characteristics by treatment group and standardized differences.

	without adjuvant treatment (n = 164)	with adjuvant treatment (n = 182)	Standardized differences observed data
**Categorical covariates: n (%)**			
**Tumor stage**			
pT1b	30 (18.3)	32 (17.6)	-0.02
pT2	107 (65.2)	118 (64.8)	-0.01
pT3	23 (14.0)	30 (16.5)	0.07
pT4	4 (2.4)	1 (0.6)	**-0.16**
unknown	0	1 (0.6)	**0.11**
**Resection status**			
R0	20 (12.2)	35 (19.2)	**0.19**
R1	122 (74.4)	134 (73.6)	-0.02
unknown	22 (13.4)	13 (7.1)	**-0.21**
**Grading**			
G1	8 (4.9)	10 (5.5)	0.03
G2	98 (59.8)	106 (59.0)	-0.03
G3	55 (33.5)	62 (34.1)	0.01
unknown	3 (1.8)	4 (2.2)	0.03
**Positive LN**			
1	84 (51.2)	59 (32.4)	**-0.39**
2	32 (19.5)	47 (25.8)	**0.15**
3	18 (11.0)	27 (14.8)	**0.12**
>3	24 (14.6)	40 (22.0)	**0.19**
unknown	6 (3.7)	9 (5.0)	0.06
**ECOG**			
0	32 (19.5)	56 (30.8)	**0.26**
1	24 (14.6)	43 (23.6)	**0.23**
2	21 (12.8)	27 (14.8)	0.06
3	13 (7.9)	6 (3.3)	**-0.20**
4	1 (0.6)	0 (0.0)	**-0.11**
unknown	73 (44.5)	50 (27.5)	**-0.36**
**Vulva surgery**			
Wide excision	19 (11.6)	9 (5.0)	**-0.24**
Partial vulvectomy	42 (25.6)	50 (27.5)	0.04
Complete vulvectomy	101 (61.6)	120 (65.9)	0.09
	2 (1.2)	3 (1.7)	0.04
**Groin surgery**			
- After initial sentinel node dissection	52 (31.7)	35 (19.2)	**-0.29**
- Primary complete groin dissection	103 (62.8)	136 (74.7)	**0.26**
- Unknown if primary or secondary	9 (5.5)	11 (6.04)	0.02
**Groin dissection**			
- Unilateral	47 (28.7)	27 (14.8)	**0.34**
- Bilateral	117 (71.3)	155 (85.2)	**0.34**
**Pelvic node dissection**	16 (9.7)	23 (12.6)	0.09
**Continuous covariates: median(range)**			
**age years**	67 (20–89)	71 (30–87)	**-0.24**
**tumor diameter mm**	35 (2–240)	35 (2.8–200)	-0.08
**depth of invasion mm**	7 (1–70)	8 (1.1–110)	-0.04
**resection margin mm**	4 (1–16)	4 (0.25–25)	**0.27**
**LN metastasis diameter mm**	15 (0.3–50)	23 (1–80)	**0.44**
**number of dissected groin LNs**	15 (1–62)	16 (1–38)	-0.03

All percentages refer to columns; ECOG Eastern Cooperative Oncology Group, LN lymph-node(s) Absolute standardized differences ≥0.10 are printed in bold type. Frequencies of missing values in continuous variables were as follows: Age 0 (0%), tumor diameter 56 (17%), depth of invasion 155 (45%), resection margin 216 (62%), lymph-node metastasis diameter 242 (70%), number of dissected groin lymph-nodes 14 (4%)

### Estimating treatment effects on disease-related and unrelated death

The effect of adjuvant therapy on the competing causes of death was computed separately in the 10 imputed data sets and then averaged over data sets using Rubin's combination rules[19]. The cause-specific hazards model was applied to consider the competition of the investigated causes of death. Using this approach, the specific events are analysed separately, treating the competing events as censored. Tests were performed two-sided with a 5% level of significance.

### Propensity Scoring

#### Identifying confounders and estimation of the PS

Confounding exists if a baseline variable correlates with the outcome and is furthermore imbalanced between the treatment groups[20]. Identification of relevant variables to be included in the PS model is a key factor for confounding control. Simulations showed that variables that are related to the outcome should be included in the model, even though they are not associated with the exposure [21]. The variance of the estimated exposure effect is decreased by this technique, without increasing bias [21]. In contrast, variables that are imbalanced with respect to the exposure can only produce bias, if they were related to the outcome. Including variables associated with the exposure but not with the outcome would increase this variance without decreasing bias [21,22]. However, the ultimate aim of propensity scoring is to balance covariates. Therefore, an iterative procedure was described by Austin (2011) [8]. In his work, he proposed to start with an initially specified propensity score model and to evaluate the resulting balance. If important systematic differences between exposure groups remain, the PS model should be modified. This procedure can be repeated until the group differences have been “reduced to an acceptable level” [8]. In the present investigation, we follow these two approaches. In an initial step, all potential confounders associated with either one of the competing endpoints were taken into account. Associations with outcome were tested using univariate cause-specific hazards models stratified across the 10 imputed data sets $X_{complete}^{\left(1\right)},\ldots ,X_{complete}^{\left(10\right)}$. After applying the PS and evaluating the balance achievement, the selection of confounders was adjusted iteratively until acceptable balance for all covariates was achieved.

The PS as defined by Rosenbaum and Rubin[1] represents the conditional probability of receiving the treatment of interest, given the variables observed at baseline. It was estimated using multivariate logistic regression of the treatment status on the confounding baseline covariates selected in the previous step. The resulting logit of the PS was then used to predict the probability of being treated[5;14].

#### Application of the propensity score

The IPTW method[7;23–25] was applied in each of the imputed data sets before averaging the results. The idea behind this method is to reweight the single individuals in the data set by the inverse probability of receiving the treatment, calculated from the PS. Thus, a sample in which the treatment assignment is independent of the distribution of measured covariates has been created[8].

Stabilized weights *w*_*i*_ for individuals i have been defined as

$$w_{i}=\frac{P}{{PS}_{i}}Z_{i}+\frac{1-P}{{1-PS}_{i}}{(1-Z}_{i})$$

[23]. The variable *Z*_*i*_ indicates the treatment status for each subject i. If subject i was treated, then *Z*_*i*_ equals 1, and 0 otherwise. *PS*_*i*_ defines the individual propensity score for patient i and P is the rate of patients receiving the treatment. A robust variance estimator was used.

For comparison, PS stratification was applied. The study sample was split up into five strata according to quintiles of the PS. Stratified Cox regressions for comparing treated and untreated groups were performed for each imputed data set and then averaged. A robust variance estimator was used. Rosenbaum and Rubin stated that five strata according to quintiles of the PS can remove 90 percent of the bias in the considered covariates[1]. If the PS was correctly specified, the treated and untreated subjects within each stratum would have similar distributions of baseline covariates and could be compared directly without bias[26].

#### Balance check

If all prognostically relevant covariates were balanced between the treatment groups the result of a univariate group comparison could be interpreted as a causal effect. The recommended way to examine if continuous variables are balanced is to compute standardized differences between treatment groups, defined as the difference between treated and untreated means of each factor, divided by the pooled standard deviation[5]. A method for factor variables is also described in Crowson et al.[5]. In this work, balance was tested in the originally measured data and in the data sets after applying the individual propensity score techniques. In the multiply imputed data, results of the balance checks were averaged across data sets using Rubin’s rules[19]. Absolute values of standardized differences <0.1 indicated sufficient balance[26].

Achieving balance across treatment groups is the goal of PS. Therefore, balance was checked after PS application. Depending on the resulting balance, the set of confounding variables was adapted and a new PS was calculated and applied.

## Software

For MI, the Stata packages "ice" and "mim" were utilized[10;27;28]. R.2.15 (The R Foundation for Statistical computing, Vienna, Austria) and Stata (StataCorp. 2015. Stata Statistical Software: Release 14. College Station, TX: StataCorp LP.) with the packages "pscore"[29] and "pbalchk"[30] were applied for PS.

## Results

Out of 346 included patients with lymph-node positive vulvar cancer and documented follow-up, 182 (53%) received adjuvant radiation therapy. Median follow-up was 16.4 months (range 0.3–163.6 months). During follow-up, 78 disease-related deaths, 17 disease-unrelated deaths and 40 deaths due to unknown reasons were observed. Median disease-free and overall survival were 15.3 months and 42.7 months, respectively. The patient characteristics as well as their association with treatment assignment are summarized in Table 1. Several differences between the treated and untreated patients were observed. Treated patients had considerably better Eastern Cooperative Oncology Group (ECOG) performance status than untreated patients, but at the same time treated patients were older and had more affected lymph-nodes with larger lymph-node metastases. Additionally, distribution of the type of groin surgery and groin dissection differed amongst the groups.

### Missing values

Of the 346 patients, only 24 (7%) were completely documented regarding the 15 considered covariates (Table 1), whereas 79 (23%) had more than three missing values. Of the 15 considered variables, only four were fully documented. Lymph-nodes metastasis diameter (70% missing) and minimum resection margin (62.4% missing) could not be considered as covariates due to their high missing rates.

### Naïve group comparison

Naïve univariate comparisons of the treated and untreated patients showed no associations between therapy and disease-related or unrelated mortality (hazard ratio (HR) 0.83, 95% confidence interval (CI): 0.53–1.29; p = 0.403 and HR 0.70, 95% CI 0.42–1.18; p = 0.177, respectively) (Table 2).

**Table 2:** Univariate associations between baseline characteristics and competing causes of death.

	Observed data				Imputed data			
	HR (95%-CI) DRD	p-value	HR (95%-CI) DOC	p-value	HR (95%-CI) DRD	p-value	HR (95%-CI) DOC	p-value
**Therapy**	0.83 (0.53–1.29)	0.403	0.70 (0.42–1.18)	0.177	0.83 (0.53–1.29)	0.403	0.70 (0.42–1.18)	0.177
**Tumor stage**								
pT2 vs pT 1b	1.43 (0.77–2.65)	0.254	2.30 (1.02–5.18)	**0.045**	1.43 (0.77–2.65)	0.254	2.30 (1.02–5.18)	**0.045**
pT3 vs pT 1b	1.66 (0.79–3.51)	0.183	2.15 (0.81–5.72)	0.126	1.66 (0.79–3.51)	0.183	2.15 (0.81–5.72)	0.126
pT 4 vs pT 1b	8.84 1.94–40.2)	**0.005**			8.84 (1.94–40.2)	**0.005**		
Unknown vs. pT 1b								
**Resection status**								
R1 vs R0	0.71 (0.38–1.32)	0.273	0.43 (0.22–0.83)	**0.012**	0.69 (0.38–1.27)	0.235	0.43 (0.22–0.82)	**0.010**
Unknown vs R0	0.86 (0.35–2.12)	0.750	1.01 (0.43–2.41)	0.979				
**Grading**								
G2 vs G1	1.27 0.45–3.55)	0.653	0.84 (0.29–2.43)	0.743	1.24 (0.44–3.48)	0.677	0.87 (0.30–2.51)	0.793
G3 vs G1	2.25 (0.80–6.38)	0.126	1.72 (0.59–4.99)	0.317	2.18 (0.77–6.18)	0.141	1.76 (0.61–5.09)	0.298
Unknown vs G1			2.75 (0.61–12.5)	0.190				
**Positive LN**								
2 vs 1	1.82 (0.94–3.53)	0.075	2.44 (1.22–4.90)	**0.012**	1.73 (0.90–3.35)	0.102	2.35 1.17–4.73)	**0.017**
3 vs 1	2.39 (1.18–4.82)	**0.015**	3.01 (1.43–6.35)	**0.004**	2.20 (1.09–4.45)	**0.029**	2.83 (1.35–5.95)	**0.006**
>3 vs 1	5.50 (2.99–10.1)	**<0.001**	2.33 (1.01–5.38)	**0.048**	5.13 (2.80–9.38)	**<0.001**	2.41 (1.03–5.64)	**0.043**
Unknown vs 1	1.84 (0.63–5.39)	0.266	1.25 (0.29–5.47)	0.766				
**ECOG**								
1 vs 0	2.95 (1.43–6.10)	**0.004**	1.44 (0.55–3.74)	0.457	1.91 (0.95–3.83)	0.068	1.52 (0.71–3.25)	0.281
2 vs 0	2.74 (1.32–5.72)	**0.007**	2.38 (1.03–5.52)	**0.044**	2.08 (1.04–4.15)	**0.038**	2.07 (0.94–4.57)	0.072
3 / 4 vs 0	2.03 (0.58–7.14)	0.268	2.42 (0.67–8.71)	0.177	1.91 (0.63–5.75)	0.252	2.97 (1.12–7.84)	**0.028**
Unknown vs 0	2.46 (1.27–4.74)	**0.007**	2.49 (1.22–5.08)	**<0.012**				
**Vulva surgery**								
Wide excision								
Partial vulvectomy	0.48 (0.21–1.08)	0.077	1.27 (0.28–5.84)	0.756	0.48 (0.21–1.08)	0.077	1.27 (0.28–5.84)	0.756
Complete vulvectomy	0.71 (0.35–1.45)	0.353	2.80 (0.68–11.5)	0.155	0.71 (0.35–1.45)	0.353	2.80 (0.68–11.5)	0.155
Exenteration	2.98 (0.80–11.1)	0.104			2.98 (0.80–11.1)	0.104		
**Groin surgery**							1	
Primary complete groin diss.								
After sentinel node diss.	0.55 (0.31–0.96)	**0.036**	1.46 (0.84–2.53)	0.179	0.52 (0.29–0.92)	**0.025**	1.43 (0.83–2.48)	0.196
Unknown			0.60 (0.14–2.51)	0.486				
**Groin dissection**								
Bi- vs unilateral	1.20 (0.67–2.14)	0.537	0.48 (0.28–0.82)	**0.007**	1.20 (0.67–2.14)	0.537	0.48 (0.28–0.82)	**0.007**
**Pelvic node dissection**	1.00 (0.52–1.95)	0.997	0.68 (0.27–1.71)	0.415	1.00 (0.52–1.95)	0.997	0.68 (0.27–1.71)	0.415
**Age (years)**	1.02 (1.01–1.04)	**0.012**	1.06 (1.03–1.09)	**<0.001**	1.02 (1.01–1.04)	**0.012**	1.06 (1.03–1.09)	**<0.001**
**Tumor diameter mm**	1.00 (1.00–1.01)	0.087	1.00 1.00–1.01)	0.264	1.01 (1.00–1.01)	0.074	1.00 (1.00–1.01)	0.488
**Depth of invasion mm**	1.01 (1.00–1.03)	0.061	1.01 (1.00–1.03)	0.099	1.01 (1.00–1.02)	0.190	1.01 (0.99–1.03)	0.291
**Number of diss. groin LNs**	0.98 (0.96–1.00)	0.112	1.01 (0.98–1.04)	0.523	0.98 (0.95–1.01)	0.121	1.01 (0.98–1.04)	0.628

DRD disease-related death, DOC death from other / unknown cause, ECOG Eastern Cooperative Oncology Group, HR hazard ratio, IPTW inverse probability of treatment weighting

### Selection of confounders for computing the PS

Associations with disease-related death were found for the variables tumor stage, ECOG, number of affected nodes, type of groin surgery and age in the original data set as well as in the imputed data. Tumor stage, resection status, ECOG, number of affected nodes, type of groin dissection (uni- / bilateral) and age were related to death from other / unknown causes in both, the original and the imputed data (Table 2). These variables also show imbalances with regards to the standardized differences (Table 3). With respect to the achieved balance the best results were obtained by considering all these potential confounders except tumor stage to compute the PS.

**Table 3:** Standardized differences to identify imbalances between treatment groups before and after imputing and inverse-probability-of-treatment-weighting.

	Standardized differences observed data	Standardized differences multiply imputed data	Standardized differences observed data IPTW	Standardized differences multiply imputed data IPTW
**Tumor stage**				
pT1b	-0.02	-0.02	**0.14**	0.08
pT2	-0.01	-0.001	-0.02	0.04
pT3	0.07	0.07	-0.05	-0.07
pT4	**-0.16**	**-0.16**	**-0.24**	**-0.20**
unknown	**0.11**		0.08	
**Resection status**				
R0	0.19	**0.17**	-0.003	0.004
R1	-0.02	**-0.17**	0.01	0.004
unknown	**-0.21**		-0.01	
**Grading**				
G1	0.03	0.03	0.02	-0.01
G2	-0.03	-0.04	-0.07	-0.05
G3	0.01	0.02	0.03	0.06
unknown	0.03		0.10	
**Positive LN**				
1	**-0.39**	**-0.39**	0.02	0.03
2	**0.15**	**0.17**	-0.01	-0.01
3	**0.12**	**0.13**	-0.002	-0.003
>3	**0.19**	**0.19**	-0.02	-0.01
unknown	0.06		0.01	
**ECOG**				
0	**0.26**	**0.16**	0.03	0.02
1	**0.23**	**0.17**	-0.05	-0.03
2	0.06	-0.05	-0.01	-0.01
3 / 4	**-0.20**	**-0.43**	-0.01	0.03
unknown	**-0.36**		0.02	
**Vulva surgery**				
Wide excision	**-0.24**	**-0.24**	**0.18**	**-0.20**
Partial vulvectomy	0.04	0.04	0.10	**0.12**
Complete vulvectomy	0.09	0.09	0.02	0.01
Exenteration	0.04	0.04	-0.004	0.001
**Groin surgery**				
- After initial sentinel node dissection	**-0.29**	**-0.27**	0.02	-0.01
- Primary complete groin dissection	**0.26**	**0.27**	-0.01	-0.01
- Unknown if primary or secondary	0.02		-0.01	
**Groin dissection**				
Bilateral vs. Unilateral	**0.34**	**0.34**	-0.01	-0.04
**Pelvic node dissection**	0.09	0.09	-0.07	0.004
**age years**	**-0.24**	**-0.24**	0.02	0.01
**tumor diameter mm**	-0.09	-0.12	**-0.20**	**-0.26**
**depth of invasion mm**	-0.04	-0.04	**-0.16**	-0.10
**number of dissected groin LNs**	-0.03	-0.04	**-0.20**	**-0.19**

LN lymph-node(s), IPTW inverse probability of treatment weighting; Standardized differences for imbalanced variables (absolute standardized differences ≥0.10) are printed in bold.

### Inverse-probability-of-treatment-weighting (IPTW)

Weighting the data according to the inverse probability of treatment resulted in predominantly balanced confounding variables (Table 3). Estimated hazard ratios after MI and IPTW for DRD were HR 0.69; 95% CI: 0.43–1.12; p = 0.135 and for DOC HR 0.73; 95% CI: 0.42–1.27; p = 0.269, respectively (Table 4).

**Table 4:** Results after propensity scoring using the potential confounders age, resection status, ECOG, number of affected nodes, type of vulva surgery and groin dissection.

	Observed data				Imputed data			
	HR (95%-CI) DRD	p-value	HR (95%-CI) DOC	p-value	HR (95%-CI) DRD	p-value	HR (95%-CI) DOC	p-value
Therapy IPTW	0.72(0.44–1.17)	0.190	0.83(0.48–1.45)	0.513	0.69 (0.43–1.12)	0.135	0.73 (0.42–1.27)	0.269
Therapy Stratification	0.93 (0.46–1.87)	0.845	1.17 (0.51–2.67)	0.708	0.66 (0.40–1.09)	0.103	0.75 (0.41–1.36)	0.337

DRD disease-related death, DOC death from other / unknown cause, HR hazard ratio, IPTW inverse probability of treatment weighting

### PS stratification

Based on the quintiles of the PS, the data set was stratified into four groups with 69 patients and one group with 70 patients. Effect estimates pooled across strata and combined from the multiply imputed data sets were HR: 0.66; 95% CI: 0.40–1.09; p = 0.103 for DRD and HR: 0.75; 95% CI: 0.41–1.36; p = 0.337 for DOC, respectively (Table 4).

## Discussion

In this study, MI followed by PS was applied to estimate the causal effect of radiation therapy in lymph-node positive vulvar cancer on competing causes of death using data from the AGO-CaRE 1 study[17].

In detail, ten complete data sets were generated using MI by chained equation (MICE), stratified by treatment allocation[10;15;16]. Then, confounders to include in the PS calculation were identified by testing univariate associations between baseline covariates and outcomes, stratified across the multiple complete data sets. Thirdly, the PS was computed for each subject. In a fourth step, PS was applied using IPTW and PS stratification[6;8;19;26;31;32]. With IPTW, each patient was weighted according to her PS value. Stratification entailed splitting each data set according to quintiles of the PS and performing analyses stratified over groups. The achieved balance of baseline covariates before and after MI and IPTW was evaluated by standardized differences. The cause-specific hazards model was used to evaluate associations between treatment allocation and the competing causes of death. Results were estimated within each of the imputed data sets and then averaged. This approach is comparable to the 'Within approach' from Mitra and Reiter (2012), who applied PS matching after MI[33]. In contrast, other approaches to overcome the problem of missing values in PS estimation have been studied[9;11;12]. For example, Qu and Lipkovich (2009) proposed an adaptation including indicators of missing data patterns in the PS model. This technique may reduce bias when data are not missing at random[11].

The results from both applied PS methods after MI were very similar and also comparable to those from the naïve group comparison without MI and PS. All approaches agree in showing no associations, but slight tendencies towards improved disease-related survival in patients receiving radiation therapy (Table 4).

The two other established PS methods, PS matching and PS covariate adjustment, were not appropriate in this example. PS matching entails assigning matched sets of treated and untreated patients, sharing a similar PS value. Various techniques are available to select one or more untreated subjects to match each treated subject [2;8;26;31;32;34–38]. However, all PS matching approaches require the group of untreated patients to be large enough (two- to threefold larger than the group of treated subjects) to provide acceptable matching partners[32]. In the AGO-CaRE 1 data, the number of treated patients exceeded the number of controls. In such situations, matching would either result in heterogeneous matched pairs or in a small number of matched pairs, omitting a significant amount of treated or untreated subjects for which no appropriate matching partner could be found. Therefore, PS matching was not implemented in this work. With PS covariate adjustment, the PS is included as adjusting covariate in a Cox proportional hazards model, where the outcome is regressed on the treatment variable. There is currently no consensus whether there is a benefit of this method, compared to performing a multivariate regression model adjusted for the confounding variables[39]. Furthermore, differences in covariate variances between treated and untreated patients can cause difficulties. In such cases, D'Agostino (1998)[32] advises against this method, which was therefore not applied in the present work.

The general purpose of the PS method is to reduce imbalances in outcome-related variables. Most imbalances that were present in originally observed and imputed data were cured after IPTW. The tumor stage, the type of vulva surgery, tumor diameter and the number of dissected groin lymph-nodes were still imbalanced in the multiply imputed data. However, these variables (except tumor stage) had no association with the outcome (Table 2) and therefore do not bias the results.

The validity of the results is limited by the assumptions inherent to the methods used. MI requires that the missing values are missing at random, which led to a similar distribution of baseline variables (Table 3) and similar univariate associations between baseline variables and outcome (Table 2) in the originally observed and the multiply imputed data. A general assumption in all PS methods is the presumption of no unmeasured confounders. Confounders that are not accounted for because they are not or imperfectly measured or not measurable can still bear a bias. In the present example, psychological factors and quality-of-life aspects were not considered and may therefore bear the risk of unmeasured confounding.

In conclusion, the points to consider in our PS application were:

1. Missing values can be a problem in propensity score analysis. Different methods like MI, as applied here, or the use of an missing values pattern indicator[9;11;12] are available. In our example, results from a complete case analysis did not differ much from PS after MI.
2. Different propensity score methods are established, like matching, stratification or IPTW, each providing even more options to choose from. The IPTW method yields an averaged treatment effect of all subjects, in contrast to most matching methods, which calculate the averaged treatment effect of the treated patients. Further, if the groups have similar size, the IPTW method performs well[9].
3. The set of confounders to include in the PS have to be chosen carefully. The main goal of all PS methods is however to obtain balance in the variables considered to be “important” in the analysis.
4. For computing the PS, a logistic regression model is the established method. However, there are also other ways including boosting or CART models[40].

## References

[1] Rosenbaum P, Rubin D. The central role of the propensity score in observational studies for causal effects. Biometrika 1983;70:41–55.

[2] Rosenbaum P, Schenck LA. Constructing a control group using multivariate matched sampling methods that incorporate the propensity score. American Statistician 39, 33–38. 1985.

[3] Austin PC. Balance diagnostics for comparing the distribution of baseline covariates between treatment groups in propensity-score matched samples. Stat Med 2009 11 10;28(25):3083–107. 10.1002/sim.3697 19757444 PMC3472075

[4] Brookhart MA, Wyss R, Layton JB, Sturmer T. Propensity score methods for confounding control in nonexperimental research. Circ Cardiovasc Qual Outcomes 2013 9 1;6(5):604–11. 10.1161/CIRCOUTCOMES.113.000359 24021692 PMC4032088

[5] Crowson CS, Schenck LA, Green AB, Atkinson EJ, Therneau TM. The Basics of Propensity Scoring and Marginal Structural Models. Department of Health Sciences Research, Mayo Clinic Rochester, Minnesota; 2013 8 1.

[6] Rosenbaum P, Schenck LA. Reducing bias in observational studies using subclassification on the propensity score. Journal of the American Statistical Association 79, 516–524. 2014.

[7] Rosenbaum P. Model-based direct adjustment. The Journal of the American Statistician 82, 387–394. 1987.

[8] Austin PC. An Introduction to Propensity Score Methods for Reducing the Effects of Confounding in Observational Studies. Multivariate Behav Res 2011 5;46(3):399–424. 10.1080/00273171.2011.568786 21818162 PMC3144483

[9] Stuart E. Matching Methods for Causal Inference: A Review and a Look Forward. Statistical Science 2010;25(1):1–21. 10.1214/09-STS313 20871802 PMC2943670

[10] Lunt M. A Guide to Imputing Missing Data with Stata—Revision: 1.4. 2011.

[11] Qu Y, Lipkovich I. Propensity score estimation with missing values using a multiple imputation missingness pattern (MIMP) approach. Stat Med 2009 4 30;28(9):1402–14. 10.1002/sim.3549 19222021

[12] Seaman SWI. Inverse Probability Weighting with Missing Predictors of Treatment Assignment or Missingness. Communication in Statistics—Theory and Methods 2014;43(16):3499–515.

[13] Royston P, White IR. Multiple Imputation by Chained Equations (MICE): Implementation in Stata. Journal of Statistical Software 2011;45(4):1–20.

[14] Rubin DB. Multiple Imputation for Nonresponse in Surveys. New York: Wiley; 1987.

[15] Van Buuren S., Boshuizen HC, Knook DL. Multiple imputation of missing blood pressure covariates in survival analysis. Stat Med 1999 3 30;18(6):681–94. 10204197 10.1002/(sici)1097-0258(19990330)18:6<681::aid-sim71>3.0.co;2-r

[16] Van Buuren S. Multiple imputation of discrete and continuous data by fully conditional specification. Stat Methods Med Res 2007;16:219–42. 10.1177/0962280206074463 17621469

[17] Mahner S, Jueckstock J, Hilpert F, Neuser P, Harter P, de Gregorio N, et al Impact of adjuvant therapy in lymph-node positive vulvar cancer—the AGO-CaRE 1 (Chemo- and Radiotherapy in Epithelial Vulvar Cancer) study. Journal of the National Cancer Institute 107 [3]. 2015. 10.1093/jnci/dju426 PMC4356703 25618900

[18] White IR, Royston P, Wood AM. Multiple imputation using chained equations: Issues and guidance for practice. Stat Med 2011 2 20;30(4):377–99. 10.1002/sim.4067 21225900

[19] Rubin DB. Propensity score methods. Am J Ophthalmol 2010 1;149(1):7–9. 10.1016/j.ajo.2009.08.024 20103037

[20] Mertens BJ, Datta S, Brand R, Peul W. Causal effect estimation strategies in a longitudinal study with complex time-varying confounders: A tutorial. Stat Methods Med Res 2014 8 20. 10.1177/0962280214545529 25147227

[21] Brookhart MA, Schneeweis S, Rothman KJ, Glynn RJ, Avorn J, Sturmer T. Variable selection for propensity score models. Am J Epidemiol. 2006 6 15;163(12):1149–56. 10.1093/aje/kwj149 16624967 PMC1513192

[22] Greenland S, Pearl J. Adjustments and their Consequences—Collapsibility Analysis using Graphical Models. International Statistical Review 2011, 79, 3, 401–426

[23] Robins JM, Hernan MA, Brumback B. Marginal structural models and causal inference in epidemiology. Epidemiology 2000 9;11(5):550–60. 10955408 10.1097/00001648-200009000-00011

[24] Hernan MA, Brumback B, Robins JM. Marginal structural models to estimate the causal effect of zidovudine on the survival of HIV-positive men. Epidemiology 2000 9;11(5):561–70. 10955409 10.1097/00001648-200009000-00012

[25] Lunceford JK, Davidian M. Stratification and weighting via the propensity score in estimation of causal treatment effects: a comparative study. Stat Med 2004 10 15;23(19):2937–60. 10.1002/sim.1903 15351954

[26] Austin PC. A Tutorial and Case Study in Propensity Score Analysis: An Application to Estimating the Effect of In-Hospital Smoking Cessation Counseling on Mortality. Multivariate Behav Res 2011;46(1):119–51. 10.1080/00273171.2011.540480 22287812 PMC3266945

[27] Royston P, Carlin JB, White IR. Multiple imputation of missing values: New features for mim. The Stata Journal 2009;9(2):252–64.

[28] Royston P. Multiple imputation of missing values: update. The Stata Journal 2005;5(2):1–14.

[29] Becker SO, Ichino A. Estimation of average treatment effects based on propensity scores. The Stata Journal 2[4], 358–377. 2002.

[30] PBALCHK: Checking Covariate Balance [computer program]. 2015.

[31] Austin PC. The use of propensity score methods with survival or time-to-event outcomes: reporting measures of effect similar to those used in randomized experiments. Stat Med 2014 3 30;33(7):1242–58. 10.1002/sim.5984 24122911 PMC4285179

[32] D'Agostino RB Jr. Propensity score methods for bias reduction in the comparison of a treatment to a non-randomized control group. Stat Med 1998 10 15;17(19):2265–81. 9802183 10.1002/(sici)1097-0258(19981015)17:19<2265::aid-sim918>3.0.co;2-b

[33] Mitra R, Reiter JP. A comparison of two methods of estimating propensity scores after multiple imputation. Stat Methods Med Res 2012 6 11. 10.1177/0962280212445945 22687877

[34] PSMATCH2: Stata module to perform full Mahalanobis and propensity score matching, common support graphing, and covariate imbalance testing [computer program]. Version version 4.0.10 10feb2014 2014.

[35] Dehejia RH, Wahba S. Propensity score matching methods for nonexperimental causal studies. RevEcon Stat 84, 151 2012.

[36] Dehejia RH, Wahba S. Causal effects in nonexperimental studies: re-evaluation of the evaluation of training programs. Journal of the American Statistical Association 94, 1043–1062. 1999.

[37] Baser O. Too much ado about propensity score models? Comparing methods of propensity score matching. Value Health 2006 11;9(6):377–85. 10.1111/j.1524-4733.2006.00130.x 17076868

[38] Rubin DB. Bias Reduction Using Mahalanobis-Metric Matching. Biometrics 36, 293–298. 1980.

[39] Rubin DB. Using multivariate matched sampling and regression adjustment to control bias in observational studies. Journal of the American Statistical Association 74, 318–324. 1979.

[40] Westreich D, Lessler J, Funk MJ. Propensity score estimation: neural networks, support vector machines, decision trees (CART), and meta-classifiers as alternatives to logistic regression. J Clin Epidemiol 2010 8;63(8):826–33. 10.1016/j.jclinepi.2009.11.020 20630332 PMC2907172

